# The Intracellular Free Zinc Level Is Vital for Treg Function and a Feasible Tool to Discriminate between Treg and Activated Th Cells

**DOI:** 10.3390/ijms19113575

**Published:** 2018-11-13

**Authors:** Martina Maywald, Fudi Wang, Lothar Rink

**Affiliations:** 1 Institute of Immunology, RWTH Aachen University Hospital, Pauwelsstr. 30, 52074 Aachen, Germany; martina.maywald@rwth-aachen.de; 2 Department of Nutrition, Precision Nutrition Innovation Center, School of Public Health, Zhengzhou University, 100 Science Road, Zhengzhou 450001, China; FWang@zju.edu.cn

**Keywords:** Treg, intracellular zinc level, cell differentiation, interferon (IFN)-γ

## Abstract

The intracellular free zinc level and zinc distribution are important for cellular function. Both are highly variable and are altered due to intrinsic zinc pool fluctuation via buffering and muffling reactions. Multiple autoimmune diseases are associated with pathologically changed zinc levels, which provoke altered signal transduction leading to changed immune responses, cell differentiation, and function. For instance, immunological tolerance can be impaired, causing autoimmune diseases because of a malfunction of regulatory T cells (Tregs). We investigated the intracellular free zinc concentration of resting and activated T helper (Th) cells and Tregs in an allogeneic graft versus host disease model using fluorescence-activated cell sorting (FACS) analysis and enlightened cell function under nontoxic zinc concentrations and zinc deficiency by detecting cytokine secretion via enzyme-linked immunosorbent assay (ELISA). We exhibited for the first time that Tregs could be explicitly discriminated from other Th cell subsets using significantly increased intracellular free zinc levels. Moreover, the intracellular free zinc level was essential in maintaining the Treg phenotype and function, since zinc deficiency favored the pro-inflammatory immune response. Therefore, we hypothesize that the intracellular free zinc level in Th cells is essential in guaranteeing proper cellular function and can be used to discriminate Tregs from other Th cell subsets.

## 1. Introduction

Since the early 1960s, zinc has been discovered to be essential in humans. Proper zinc homeostasis is indispensable to an adequate immune response, since zinc deficiency is known to increase the incidence of bacterial or viral infections, the development of cancer, graft rejection, and autoimmune diseases [1,2,3]. In line with that, studies have uncovered a correlation between patients suffering from multiple sclerosis (MS) and lowered plasma zinc levels, as well as lower zinc concentrations in chronic MS lesions [2,4,5]. Additionally, dietary deficiency has been shown to be associated with the risk and development of pathogenic pro-inflammatory T cells in an MS mice model [6,7]. It is well known that especially T cells are affected by altered zinc levels, as shown in T cell development, maturation, activation, and differentiation [8,9,10]. Thus, zinc deficiency can lead to reduced proliferation, reduced response to mitogens, thymic atrophy, a selective decrease in Th cells (CD4-expressing T cells), and overall altered T cell differentiation [11,12,13,14].

Interestingly, zinc supplementation in nontoxic doses is well known to beneficially influence malfunctioning immunological processes. For instance, the zinc deficiency-induced shift of the T helper (Th) subset differentiation from Th1 to Th2 can be reversed by zinc supplementation. Th2-mediated allergic immunoreactions are reduced, and the protecting immunoreaction against invading bacteria or viruses is restored, thus re-establishing proper immune function [15]. Besides Th1 and Th2 differentiation, regulatory T cell (Treg) differentiation and stability can also be modulated by zinc in allogeneic immunoreactions [16,17,18]. This is of fundamental importance, since Treg therapy in clinical trials seems to be promising in treating different diseases like organ-specific and systemic autoimmune diseases and in graft acceptance in transplantation [19,20,21,22].

Although graft rejections and the development of autoimmune diseases are associated with a disturbed zinc homeostasis, a suboptimal zinc status in patients cannot easily be determined or correlated with diseases due to the lack of clinical signs and reliable biomarkers. Until now, no specific and reliable biomarker of zinc status in men has been found [23]. This is due to the effects of multiple confounders such as infection, inflammatory conditions, and time and composition of the last meal [24]. Hence, plasma zinc level can stay normal while the intracellular free zinc level may change, leading to altered signal transduction and cell function. 

The intracellular free zinc level has been recognized as important for T cell activation because zinc acts as a second messenger in signal transduction [25]. A modest increase can have profound effects on signaling pathways originating from the T cell receptor (TCR) [26], cytokine receptors [27,28,29], and Toll-like receptors (TLRs) [30]. Raised zinc concentrations inhibit the activity of intracellular kinases [31,32,33] and phosphatases [34,35] that in turn affect signal transducers and activators of transcription (STAT) and mitogen-activated protein kinase (MAPK) signaling networks and the associated transcription factor activity. Therefore, regulating the intracellular free zinc level offers the potential to influence the development of Th cell effector function and to prevent diseases arising from aberrant Th cell activity [15,36].

Hence, this study investigated the intracellular free zinc level of Tregs and other Th cells as well as the influence of zinc supplementation or zinc deficiency on an in vitro graft versus host disease (GVHD) model, called a mixed lymphocyte culture (MLCs). In MLCs, T cells of two individuals are mixed and co-cultured. Due to the expression of specific major histocompatibility complex (MHC) molecules on the cells’ surface in each individual, the other MHC molecules are recognized as foreign and an allogeneic immunoreaction is triggered, which is measurable by T cell proliferation and pro-inflammatory cytokine production [37]. For years, MLCs has been commonly used to simulate allogeneic reactions in vitro, as observed in GVHD [38,39]. 

In this study, we uncovered for the first time that Tregs and other Th cell subsets can be discriminated between using distinct intracellular free zinc levels. Moreover, Treg function was drastically impaired under zinc deficiency, whereas zinc supplementation strengthened Treg function and stability. Indeed, zinc level as a biomarker in serum is not reliable to predict disease progression and outcome. However, intracellular free zinc might be useful in discriminating Tregs. In summary, proper zinc homeostasis is critical for adequate pro-tolerogenic and pro-inflammatory immune reactions and needs to be controlled carefully. 

## 2. Results

### 2.1. Regulatory T Cells Could Be Identified by Elevated Intracellular Free Zinc Level

**Figure 1:**
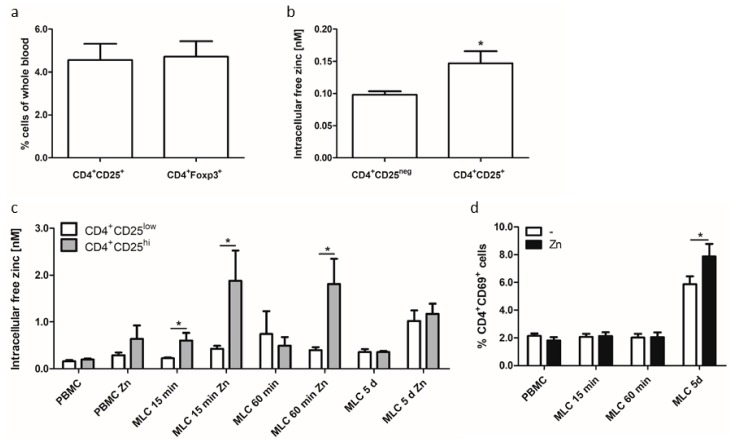
Intracellular free zinc level substantially increased in allogeneic activated regulatory T cells (Tregs). (**a,b**) Whole blood was taken from healthy volunteers and parameters were measured directly without delay or separation. (**a**) Non-activated whole blood samples were analyzed regarding cellular percentage of CD4^+^CD25^+^ and CD4^+^FOXP3^+^ expressing Tregs. (**b**) Intracellular free zinc level is displayed for CD4^+^CD25^+^ Tregs and CD4^+^CD25^neg^ T cells. (**c,d**) 2 × 10^6^ peripheral blood mononuclear cells (PBMCs)/mL were pre-incubated with 50 µM zinc (15 min) or remained untreated. Subsequently, mixed lymphocyte cultures (MLCs) were generated for 15 min, 60 min, and five days. (**c**) Measurement of the free intracellular zinc level was performed by Fluozin3-AM in gated Th cell populations: CD4^+^CD25^low^ T cells (white bars) and CD4^+^CD25^hi^ T cells (grey bars). (**d**) Activated (CD4^+^CD69^+^) Th cells in PBMCs and MLCs are displayed. (^+^: positive, expression, ^low^: low expression, ^hi^: high expression,^ neg^: negative, no expression) All data are shown as means + SEM (standard error of mean) of *n* = 4–6 independent experiments (* *p* < 0.05, Student’s *t*-test).

So far, it is known that several signaling pathways are affected by altered intracellular free zinc levels subsequently leading to changed cellular functions and immune responses. This is why the intracellular free zinc level was investigated in resting (peripheral blood mononuclear cells (PBMCs)) and allogeneic activated T cells (using MLCs) in this study. Additionally, we subdivided T cell populations in MLCs in CD4^+^CD25^low^-expressing T cells and regulatory T cells that are defined by a high expression of the interleukin (IL)-2 receptor α-chain (CD25) [40] and are thus referred to as CD4^+^CD25^hi^-expressing T cells (Figure 1). The Treg phenotype marker FOXP3 could not be used in this model because for intracellular zinc staining, vital and non-permeabilized cells are necessary. In contrast, for intracellular transcription factor staining, cells need to be permeabilized, making simultaneous zinc measurement impossible, since zinc is released from cells during permeabilization. Due to this fact, the percentage of CD4^+^CD25^+ ^and CD4^+^FOXP3^+^ Tregs was analyzed in non-activated whole blood samples (Figure 1a). Similar amounts of both cellular subsets were found, making CD25 staining an adequate method for intracellular zinc determination in Treg. Interestingly, the intracellular free zinc level in CD25^+^ Tregs was significantly elevated compared to other Th cell subsets negative for CD25 expression (CD4^+^CD25^neg^) (Figure 1b). This indicated that intracellular zinc could be used for in vivo measurement.

The analysis of isolated PBMCs and allogeneic activated MLC cells uncovered in non-activated PBMCs a slight increase in the free intracellular zinc level in CD4^+^CD25^low^ and CD4^+^CD25^hi^-expressing T cells after zinc pretreatment compared to the untreated control. In comparison to non-activated PBMCs, allogenic activated T cells in MLCs showed an increased free intracellular zinc level in CD4^+^CD25^hi^ Tregs after 15 and 60 min. By comparing the free intracellular zinc level of CD4^+^CD25^low^ and CD4^+^CD25^hi^-expressing T cells in MLCs after zinc supplementation, we interestingly uncovered a significant elevation in CD4^+^CD25^hi^-expressing T cells after 15 and 60 min. In contrast, the free intracellular zinc level in CD4^+^CD25^low^ activated T cells remained unaffected and was comparable to non-activated PBMCs.

Long-term cellular activation for five days in MLCs displayed comparable free intracellular zinc levels in Tregs and other activated T cells and was comparable to free intracellular zinc levels detected in zinc-supplemented non-activated PBMCs. This might be due to a significantly altered amount of activated (CD4^+^CD69^+^) T cells (Figure 1d), or due to the in vitro system, since freshly isolated Tregs in whole blood showed a higher zinc content (Figure 1b) in contrast to PBMCs (Figure 1c).

In summary, cellular activation, as shown in the MLC experiments, provoked a slight elevation of the intracellular free zinc level compared to non-activated PBMCs. Moreover, the intracellular free zinc level was significantly elevated in Tregs (CD4^+^CD25^hi^) compared to other Th cells (CD4^+^CD25^low^) in an early phase of activation (15 min and 60 min). This led to the hypothesis that the zinc concentration prominent before and during cellular activation is highly important for specific cellular function. Moreover, for adequate function, Tregs seemed to demand higher amounts of intracellular zinc compared to activated Th cells. This hypothesis is based on the requirement of IL-2 for Treg survival [40] and on the supportive impact of zinc on IL-2 signaling [27,28]. Thus, the intracellular free zinc level in activated T cells might be a feasible tool in discriminating between Treg and Th cells.

### 2.2. The Intracellular Free Zinc Level Controlled T Cell Differentiation and Pro-inflammatory Cytokine Secretion

**Figure 2:**
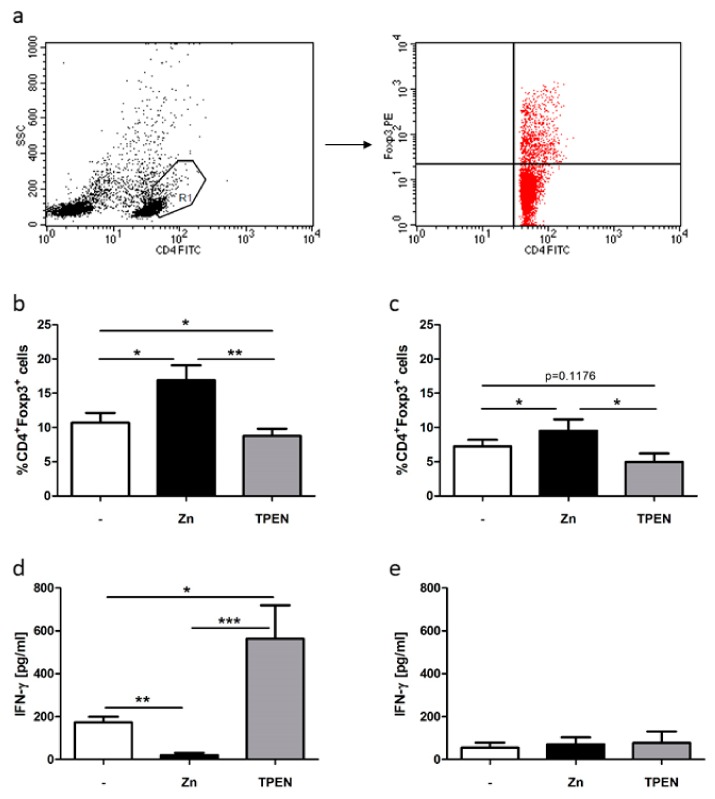
Zinc deficiency and zinc supplementation adversely influenced Treg differentiation in MLCs. Here, 2 × 10^6^ PBMCs/mL remained untreated or were pre-incubated with 50 µM zinc (black bars) or 1.5 µM *N,N,N′,N′*-Tetrakis(2-pyridylmethyl)ethylenediamine (TPEN) (grey bars) for 15 min. MLCs were generated for five days. (**a**) One representative dot blot of gated (polygon, R1) viable CD4^+^ activated T cell blasts is displayed showing side scatter (SSC) and CD4-FITC (fluoresceinisothiocyanat) staining. Gating procedure was established in reference [16]. (**b**) Only cells of R1 (red dots) are displayed and were analyzed regarding CD4-FITC and FOXP3-PE (phycoerythrin) staining. These Tregs were calculated using FACS analysis in MLCs (activated T cells) and (**c**) PBMCs (resting T cells). The concentration of the pro-inflammatory cytokine interferon (IFN)-γ was measured in (**d**) MLCs and (**e**) PBMCs. All data are shown as means + SEM of *n* = 6 independent experiments (* *p* < 0.05, ** *p* < 0.01, *** *p* < 0.001, Student’s *t*-test).

Zinc signals are important in Treg function and are crucial to T cell differentiation, as exhibited in former studies that showed induction and stabilization of FOXP3 expression due to zinc supplementation [16,36]. This study investigated whether the free intracellular zinc level of Th cells before and during activation is important for cell differentiation in the Treg subpopulation. In this regard, the available zinc level before MLC generation was manipulated through zinc supplementation in nontoxic doses (50 µM) or by zinc chelation using *N,N,N′,N′*-Tetrakis(2-pyridylmethyl)ethylenediamine (TPEN) in nontoxic doses (1.5 µM). Zinc supplementation significantly induced CD4^+^FOXP3^+^ Tregs in MLCs and PBMCs (Figure 2b,c), whereas TPEN treatment significantly reduced Treg induction in MLCs (Figure 2b).

Since the allogeneic immunoreaction in MLCs can be characterized through measurement of pro-inflammatory cytokine production, interferon (IFN)-γ secretion was analyzed in activated T cells (MLCs) and resting T cells (PBMCs) (Figure 2c). Zinc supplementation significantly dampened the alloreaction, whereas zinc deficiency negatively influenced the allogeneic immunoreaction in MLCs. In contrast, no difference compared to the untreated control was observed in PBMCs (Figure 2e).

Thus, these results indicate that zinc deficiency adversely influenced Treg differentiation in this allogeneic immunoreaction, whereas zinc supplementation induced Treg differentiation. Consequently, the free intracellular zinc level highly influenced T cell-mediated immune responses. 

## 3. Discussion

A well-operating immune system is highly dependent on the essential trace element zinc. Recent studies have uncovered zinc deficiency to be a very common phenomenon affecting approximately two billion people worldwide. Zinc deficiency is identified as a major contributor to the burden of disease in developing countries, but also in industrial countries, especially in the elderly population [41]. Unfortunately, a suboptimal zinc status in men cannot be easily diagnosed because of a lack of particular clinical symptoms and because of a lack of a specific and reliable biomarker. So far, zinc status has been characterized through the quantification of zinc concentration using, among others, serum and plasma zinc and urinary zinc excretion. However, zinc status in men is also impacted by numerous factors such as immune status, infections and inflammatory conditions, diet, and absorption and recovery via the gastrointestinal tract and kidneys [23]. This is why there is an ongoing need for the discovery of a reliable biomarker of zinc status in men to guarantee an adequate assessment of health status. 

This is especially important regarding autoimmune diseases, since a number of them are associated with disturbed zinc homeostasis, such as rheumatoid arthritis, multiple sclerosis, systemic lupus erythematosus, and colitis [4,42,43,44]. Since T cell maturation, differentiation, and function is reliant on adequate zinc status [32,45,46], this connection is likely to be one reason for the development or progression of autoimmune diseases. 

In line with this, in this study we uncovered for the first time that the intracellular free zinc level in CD4^+^CD25^hi^-expressing Tregs is significantly higher compared to CD4^+^CD25^low^-expressing Th cells. Thus, the intracellular free zinc level could be used to discriminate Tregs from other Th cell subsets. Moreover, Tregs seemed to require a higher intracellular free zinc level for adequate function due to their IL-2 dependence [40]. The intracellular free zinc level in T cells should hence be considered as potential evidence of T cell function, primarily in regard to Tregs, since the intracellular free zinc level in T cells is crucial for appropriate differentiation and function [10,45]. Although this is not a biomarker for the zinc status of patients it might be a marker for Tregs.

In agreement with this, former studies have found that zinc supplementation in nontoxic doses in MLCs in vitro ameliorated the alloreaction without affecting the immune response toward neoantigens [46,47]. Other studies have found beneficial effects of zinc supplementation in allogeneic transplantation [13,39]. Herein, the allograft rejection was reduced by zinc supplementation in a dose-dependent manner. Moreover, the graft function could be preserved due to zinc supply [39]. The authors suggested, among other reasons, that zinc-related reduced pro-inflammatory cytokine production was a possible reason, as we observed in our model.

More recently, we revealed that zinc supplementation favored Treg induction and stability by influencing several signaling pathways and molecular targets in vitro [16,17,18] and in vivo [15,36], as well as that Treg function was dependent on adequate zinc status since Treg function was impaired during zinc deficiency [48]. Hence, Tregs could be induced, which beneficially affected autoimmune diseases. Since clinical Treg therapy in the treatment of autoimmune diseases and transplantation is growing, there is an ongoing need for Treg generation ex vivo and in vivo, as well as for Treg purification and discrimination, which is still a challenge [49]. This is why the measurement of the intracellular free zinc level could be considered a strategy for Treg discrimination.

Lately, we and others have shown long-term zinc supplementation to be competent in inducing Tregs in allogeneic immunoreactions and autoimmune diseases, resulting in disease amelioration through stabilization of Tregs [36,47,50]. However, the immediate short-term effect of zinc supplementation and zinc deficiency during T cell priming and during the alloreaction in MLCs had not been investigated yet. In line with former findings, we uncovered comparable effects already after short-term treatment for 1 h (Figure 2). Herein, zinc supplementation significantly increased CD4^+^FOXP3^+^-expressing Tregs in MLCs (Figure 2b), as well as in non-activated T cells, as displayed regarding PBMCs (Figure 2c). Moreover, we uncovered a reduced pro-inflammatory cytokine production in MLCs (Figure 2d). In non-activated T cells, only basal cytokine production was measurable (Figure 2e). This led to the hypothesis that zinc status during the early phase of cellular activation seemed to be most important in Treg differentiation and function. This is underlined by the identification of Treg by intracellular zinc in freshly isolated T cells.

In contrast to zinc supplementation, zinc deficiency impeded Treg differentiation (Figure 2b,c) and triggered IFN-γ secretion (Figure 2d). Since the severity of the MLC alloreaction can be correlated to IFN-γ secretion [51], zinc supplementation significantly ameliorated the immune reaction, whereas zinc deficiency triggered the alloreaction in this in vitro GVHD model. Thus, not only the polarization of Th cells regarding Th1/Th2 cells was affected by zinc deficiency, but also the pro-tolerogenic immune response by Tregs, which led to an unbalanced cell-mediated immune response [25,26]. Hence, the available intracellular free zinc level during cellular activation was indispensable for Tregs. 

Treg function plays a fundamental role in adverse immune reactions and today’s transplantation medicine. The results of this study substantiate the importance of the essential trace element zinc in appropriate T cell function. Hence, zinc should be considered as a therapeutic supplement for clinical treatment of T cell-related disorders such as autoimmune diseases and allergies as well as adverse reactions due to solid organ transplantation. Since the immunomodulatory potential of zinc is prominent and zinc administration is relatively nontoxic and cheap, the capacity of zinc as a therapeutic supplement needs to be studied in more detail.

## 4. Materials and Methods 

### 4.1. Human PBMC Isolation and Generation of Mixed Lymphocyte Culture 

Heparinized peripheral venous blood from healthy volunteers was taken following informed consent and ethics committee approval (RWTH Aachen University Hospital, statement no. EK 023/05). Peripheral blood mononuclear cells were isolated as described before [16]. Cells were adjusted to a final concentration of 2 × 10^6^ cells/ml. For the generation of two-way MLCs, 2 × 10^6^ PBMCs/ml of two genetically diverse donors were pre-incubated with a medium or supplemented with 50 µM zinc sulfate or 1.5 µM TPEN for 15 min, followed by mixing at a 1:1 ratio in pyrogen-free 24-well dishes for indicated periods (VWR, Radnor, PA, USA). The final cell concentration in PBMCs and MLCs experimental setups was hence equal (2 × 10^6^ cells/ml). All incubation steps were carried out at 37 °C in a humidified 5% CO_2_ atmosphere.

### 4.2. Measurement of Intracellular Zinc Concentrations

The intracellular free zinc level was determined as described before [16]. In this experiment, 2 × 10^6^ PBMCs were loaded with 1 µM FluoZin3-AM; dissociation constant (*Kd*) = 8.9 nM, excitation/ emission (Ex/Em ~494/516 nm) in PBS at 37 °C for 30 min and were washed once with phosphate buffered saline (PBS). The maximum value (*Fmax*) was determined by using 100 µM zinc sulfate and 50 µM pyrithione, and the minimum value (*Fmin*) by using 2 µM TPEN. The fluorescence intensity of the sample (*F*) as well as *Fmin* and *Fmax* were analyzed with flow cytometry (FACS Calibur, BD Biosciences, Heidelberg, Germany). The intracellular free zinc level was calculated as follows: Kd*(*F* − *Fmin*)/(*Fmax* − *F*).

### 4.3. IFN-γ Cytokine Quantification

According to the manufacturer’s instructions, supernatants were stored at −20 °C and were only thawed once for cytokine detection. IFN-γ cytokine concentration was quantified by using OptEIA assays from BD PharMingen (Heidelberg, Germany).

### 4.4. Flow Cytometry 

For this analysis, 2 × 10^6^ cells were incubated with CD4-FITC (fluoresceinisothiocyanat) and CD25-APC (allophycocyanin) antibodies for 20 min at room temperature in the dark for cell surface staining. Intracellular FOXP3 staining was performed according to the manufacturer’s instructions (fix/perm kit, BD Biosciences, Heidelberg, Germany) by using a FOXP3-PE (phycoerythrin) antibody. The fluorescence intensity was measured by flow cytometry (FACS Calibur, BD Biosciences, Heidelberg, Germany).

### 4.5. Statistical Analysis

Statistical significance was calculated by Student’s *t*-test using GraphPad Prism software (version 5.01, La Jolla, CA, USA).

## 5. Conclusions

Adequate T cell differentiation and polarization are fundamentally important for well-balanced immune functions, since numerous diseases are associated with a malfunction of the Th cell compartment. In this regard, the intracellular free zinc level in Th cells is essential in guaranteeing proper cellular function and could be used to discriminate between Treg and other Th cell subsets.

## Abbreviations

ACP	Allophycocyanin
FITC	Fluoresceinisothiocyanat
FOXP3	Forkhead-Box-Protein P3
GVHD	Graft versus host disease
IFN	Interferon
IL	Interleukin
MLCs	Mixed lymphocyte cultures
MS	Multiple sclerosis
PBMCs	Peripheral blood mononuclear cells
PBS	Phosphate buffered saline
PE	Phycoerythrin
Th	T helper cell
TPEN	*N,N,N′,N′-*Tetrakis(2-pyridylmethyl)ethylenediamine
Treg	Regulatory T cell
